# Unveiling the creatinine-to-albumin ratio: a novel predictor of acute heart failure post-TBI

**DOI:** 10.3389/fnut.2026.1707810

**Published:** 2026-01-16

**Authors:** Sheng Chen, Xin Hong, Ailin Cheng, Jiayin Wang, Ling Jiang

**Affiliations:** 1 Nanping First Hospital Affiliated to Fujian Medical University, Nanping, China; 2 The Second Affiliated Hospital of Fujian Medical University, Quanzhou, China

**Keywords:** acute heart failure, creatinine-to-albumin ratio, logistic regression, propensity score matching, ROC analysis, traumatic brain injury

## Abstract

**Background:**

The creatinine-to-albumin ratio (CAR) has emerged as a potential biomarker for predicting acute heart failure (AHF) after traumatic brain injury (TBI) in various clinical settings. This study aimed to evaluate the association between CAR and AHF through logistic regression models and Receiver Operating Characteristic (ROC) analysis, both before and after propensity score matching (PSM), and to explore the dose–response relationship between CAR and AHF.

**Methods:**

A total of 1,899 patients were enrolled in the present study, including 1,836 TBI patients without AHF and 63 TBI patients with AHF. Logistic regression, ROC analysis, restricted cubic spline (RCS) modeling and CAR quartile-based trend tests were performed before and after PSM to assess the association between CAR and AHF. The unadjusted Model-1 and adjusted Model-2 (adjusted for gender, age, smoking, alcohol consumption, hypertension, and diabetes) were constructed prior to matching. One hundred twenty-four patients (62 patients in each group) were involved in the final analysis after PSM.

**Results:**

Before PSM, CAR was significantly associated with AHF after TBI in logistic regression (OR: 1.885, 95% CI: 1.534–2.318, *p* < 0.001), and demonstrated strong predictive performance in ROC analysis with an area under the curve (AUC) of 0.721 (*p* < 0.001). After PSM, CAR retained a significant association with AHF after TBI in logistic regression (OR: 1.597, 95% CI: 1.007–2.532, *p* = 0.047), although its ROC performance declined (AUC: 0.599, *p* = 0.050). RCS analysis revealed a nonlinear relationship, with higher CAR levels significantly increasing the odds of AHF (*p* < 0.001). Quartile-based trend analysis confirmed a positive association, with the highest CAR quartile (Q4) showing the greatest risk (OR: 5.980, *p* < 0.001 in Model-1; OR: 2.357, *p* = 0.062 in Model-2).

**Conclusion:**

The CAR is a significant and independent predictor of AHF following TBI, demonstrating a positive dose–response relationship with increasing CAR levels. These findings underscore the potential utility of CAR as a reliable biomarker for AHF following TBI.

## 1 Introduction

Traumatic brain injury (TBI) is a significant contributor to global disease burden. Acute heart failure (AHF) complicates approximately 5–10% of hospitalized cases as per large cohort studies (1). This is a large underestimate in milder cases of TBI. Moderate and severe TBI, as measured by low Glasgow coma scale (GCS), has significantly higher risk of developing AHF (2). The AHF remains a critical complication in patients with TBI, presenting significant challenges to clinical management and patient outcomes (3). The pathophysiological interplay between systemic inflammation, neurogenic stress responses, and cardiac dysfunction is well-documented in the literature (4, 5). And moreover mild TBI can lead to AHF which has been linked to post-concussion syndrome. Thus early detection is a clinical need in the population with TBI. Diagnosis of AHF after TBI is complicated by the use of commonly employed cardiac biomarkers such as B-type natriuretic peptide (BNP) and troponin. BNP is released directly from the brain after injury, leading to a high number of false positives in TBI patients (6). Troponin, on the other hand, can be elevated in cases of TBI as a result of sympathetic storm leading to neurogenic stress cardiomyopathy (7). These increases in troponin occur without underlying coronary ischemia. C-reactive protein (CRP) and CAR are not commonly used biomarkers of AHF. CRP is an acute phase reactant of inflammation while CAR is a biomarker of kidney dysfunction and malnutrition. However, identifying reliable biomarkers to predict AHF in TBI patients is crucial for early diagnosis and timely intervention (8).

In recent years, the CAR has garnered attention as a potential biomarker of systemic inflammation and metabolic derangements. The CAR is a combination of both, renal function and nutritional status, two factors known to be affected in cases of TBI. It is also directly related to cardiac preload and afterload and myocardial energy metabolism (9, 10). These two processes, along with inflammation, are deranged in AHF following TBI, which is why we believe CAR is a suitable candidate to be used as a cardiac biomarker in TBI patients and may provide incremental benefit. And that the both creatinine and the blood albumin are intrinsically linked to cardiac failure (11). Despite its growing application in various diseases, including cardiovascular conditions and critical illnesses, the predictive role of CAR in TBI-related AHF remains insufficiently explored, warranting further investigation.

The CAR reflects a combination of muscle metabolism and nutritional status, both of which are often compromised in critically ill patients (12, 13). Elevated serum creatinine levels have been associated with poor outcomes in conditions such as acute myocardial infarction and heart failure (14). Moreover, the serum albumin response reflects the body’s overall stress energy metabolism and nutrient metabolism, with a reduction in albumin levels often indicating heightened stress metabolism and malnutrition (15). In the context of TBI, where systemic inflammation and multi-organ dysfunction frequently occur, CAR may serve as a valuable biomarker for identifying patients at risk of AHF. However, the relationship between CAR and AHF in TBI patients remains insufficiently explored. Previous studies on biomarkers in TBI have predominantly focused on neurological outcomes, with limited emphasis on cardiovascular complications (16). This study aims to bridge this gap by examining the association between CAR and AHF in TBI patients using robust statistical methodologies.

In this study, logistic regression models and Propensity Score Matching (PSM) were used to explore the predictive value of CAR for AHF in TBI patients. Restricted cubic spline (RCS) analysis was employed to assess nonlinear relationships, and quartile-based trend tests provided insights into dose–response effects. This comprehensive analysis aims to clarify the role of CAR as a predictor of AHF in the TBI population. By identifying CAR as a potential biomarker, our findings could enhance early risk stratification and guide targeted interventions for TBI patients at high risk of cardiac complications.

## 2 Materials and methods

### 2.1 Patient selection and study cohort

This retrospective cohort study initially screened 2,988 TBI patients who were admitted to the hospital between September 1, 2016, and August 31, 2024. The inclusion criteria of this study were as follows: (1) TBI diagnosed by clinical and radiological presentation; (2) age ≥18 years; (3) patients admitted to the neurocritical care unit within 24 h of TBI onset; (4) complete medical records with serum creatinine and albumin levels within 48 h after admission; and (5) patients without any previous history of heart failure, chronic kidney diseases, and severe liver dysfunction. The exclusion criteria were as follows: (1) missing medical records and incomplete data, particularly serum creatinine and albumin levels; (2) Acute kidney injure (AKI) development during the hospital stay; (3) TBI onset to admission >24 h; (4) hospital stay <48 h; (5) severe extracranial injury and polytrauma; (6) history of malignancy, autoimmune disease, and other pre-existing chronic inflammatory disorders; (7) pregnancy and lactation; and (8) administration of immunosuppressive therapy or any other factors associated with protein metabolism.

**Figure 1:**
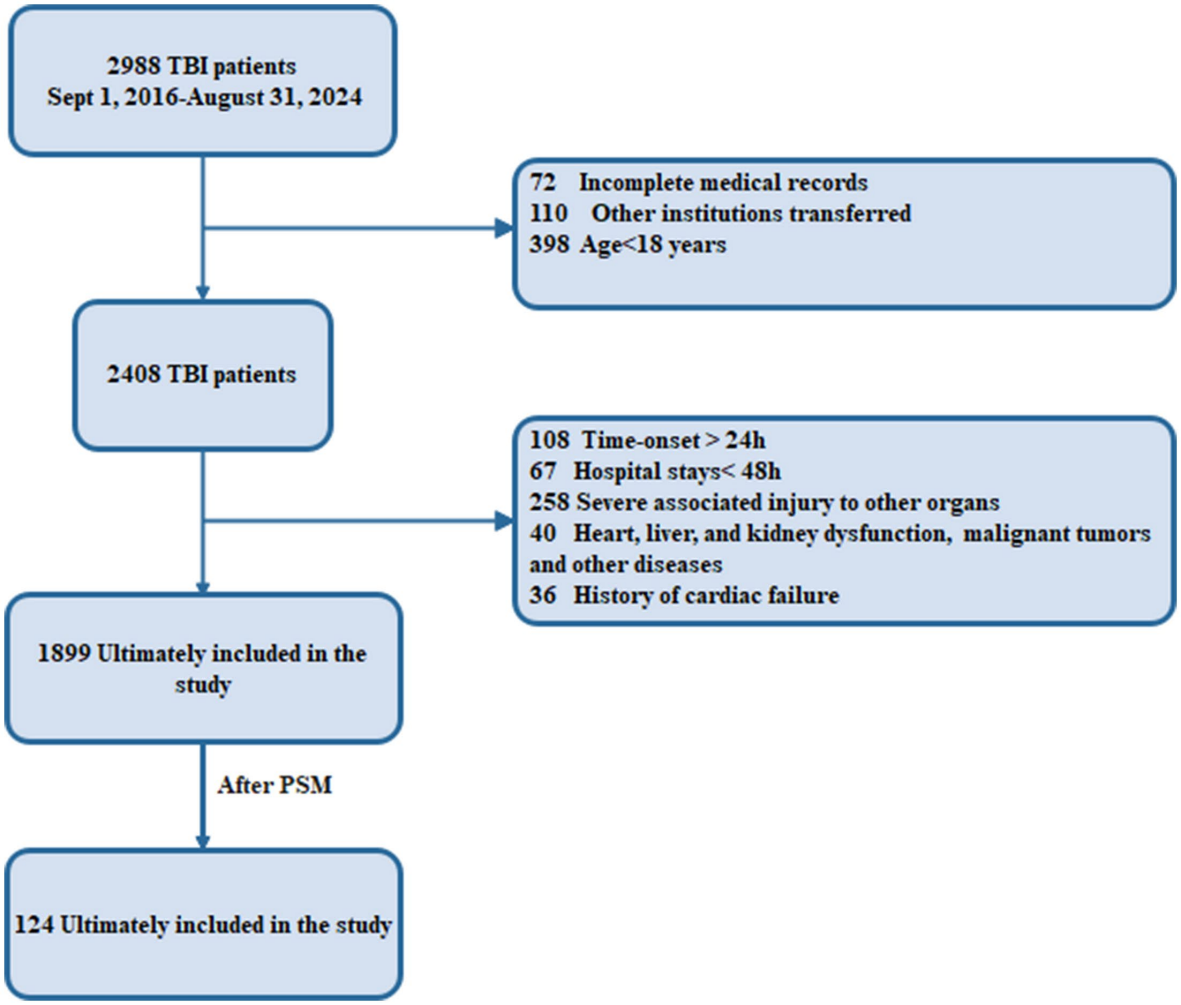
Flow diagram of patient selection process. This figure shows the screening and inclusion of TBI patients. A total of 2,988 TBI patients were screened from September 1, 2016, to August 31, 2024. After excluding incomplete medical records (*n* = 72), transfer to another hospital (*n* = 110), or age < 18 (*n* = 398), 2,408 patients were potentially eligible for further evaluation. Subsequently, additional exclusion was applied for onset time >24 h (*n* = 108), hospital stay <48 h (*n* = 67), serious associated injury to other organs (*n* = 258), heart/liver/kidney dysfunction, malignancy, or other diseases (*n* = 40), and history of cardiac failure (*n* = 36). In total, 1,899 patients were included in the main analysis. After PSM, 124 matched patients (62 pairs) were included in the final statistical analysis cohort.

The patients were chosen for the study in two phases. In the first phase, after screening, 72 patients were excluded for incomplete medical records, 110 for transfer to other institutes, and 398 for age <18 years, resulting in a population of 2,408. In the second phase, 108 patients were further excluded for admission >24 h, 67 for hospital stay <48 h, 258 for severe extracranial injuries, 40 for pre-existing organ dysfunction and malignancy, and 36 for history of cardiac failure, yielding a total of 1,899 patients for the final analysis. After propensity score matching (PSM), the total sample size was 124 patients (62 with AHF and 62 without AHF) (Figure 1). This two-phase selection of the study population ensured a homogeneous population without various confounding factors, thus increasing the clinical significance of the study results.

### 2.2 Ethical statement

This study was conducted in accordance with the Declaration of Helsinki and approved by the Institutional Ethics Committee of the Nanping First Hospital Affiliated to Fujian Medical University. Written informed consent was waived due to the retrospective nature of the study. All patient data were anonymized before analysis to ensure confidentiality.

### 2.3 Data collection

Baseline demographic, clinical, and laboratory data were collected at admission. Variables included age, gender, GCS score, time to onset, CAR, and other laboratory markers, including hemoglobin, glucose, and lipid levels. CAR was calculated by dividing serum creatinine by serum albumin, as previously described (11). According to ACC/AHA guidelines (17), patients with AHF after TBI who met the following criteria were included in this study: (1) Clinical symptoms: dyspnea, orthopnea, fatigue, or reduced exercise tolerance. (2) Physical signs: peripheral edema, jugular venous distension, and pulmonary crackles. (3) Laboratory findings: elevated natriuretic peptides (e.g., BNP > 100 pg./mL or NT-proBNP >300 pg./mL). (4) Imaging evidence: echocardiographic findings of reduced ejection fraction, left ventricular diastolic dysfunction, or pulmonary congestion.

### 2.4 Statistical analysis

All statistical analyses were conducted using SPSS (version 26.0) and R (version 4.1.3). PSM was used to minimize confounding and balance baseline characteristics between groups. Baseline demographic and clinical characteristics between the AHF group and non-AHF group were compared. The continuous variables were expressed as mean ± standard deviation and were compared using Student’s *t*-test or the Mann–Whitney *U* test for variables that were or were not normally distributed, respectively. The categorical variables were summarized as numbers (percentages) and were compared using the Chi-squared test or Fisher’s exact test where appropriate. The above statistical tests were applied to the cohorts before and after PSM, respectively. A two-sided *p*-value < 0.05 was considered statistically significant. Logistic regression models were employed to assess the association between CAR and AHF after TBI, with Model-1 unadjusted and Model-2 adjusted for gender, age, smoking, drinking, hypertension, and diabetes. Receiver Operating Characteristic (ROC) curves and RCS analysis were utilized to evaluate the predictive performance and nonlinear relationships of CAR. Quartile-based trend tests were conducted to explore dose–response effects of CAR on AHF risk. Statistical significance was defined as *p* < 0.05, and 95% confidence intervals (CI) were reported for all estimates.

## 3 Results

### 3.1 Baseline demographic and clinical characteristics before PSM

The baseline characteristics of the study population showed significant differences between patients with and without AHF after TBI (Table 1). TBI patients with AHF were significantly older (74.17 ± 12.52 years vs. 55.46 ± 16.87 years, *p* < 0.001) and had higher systolic blood pressure (143.89 ± 26.80 mmHg vs. 136.13 ± 21.30 mmHg, *p* = 0.026). These findings suggest that age and cardiovascular strain are key factors in the development of AHF among traumatic brain injury patients. Additionally, notable hematological differences were observed, with lower hemoglobin (112.50 ± 25.18 g/L vs. 125.63 ± 21.01 g/L, *p* < 0.001) and red blood cell counts (3.65 ± 0.78 × 10^12/L vs. 4.14 ± 0.72 × 10^12/L, *p* < 0.001) in the AHF group. Lymphocyte levels were also lower (1.15 ± 0.63 × 10^9/L vs. 1.36 ± 0.66 × 10^9/L, *p* = 0.011), suggesting potential immune suppression or inflammation in this subgroup.

**Table 1:** Baseline demographic and clinical characteristics of the study population before PSM.

Variable	Overall	Non-AHF	AHF	*p*-value
	*N* = 1,899	*N* = 1,836	*N* = 63	
Age, mean (±sd)	56.08 (±17.07)	55.46 (±16.87)	74.17 (±12.52)	<0.001
Time_onset, mean (±sd)	291.55 (±2,096.63)	292.24 (±2,125.91)	271.43 (±898.80)	0.867
R, mean (±sd)	19.65 (±3.26)	19.63 (±3.23)	20.06 (±4.21)	0.423
T, mean (±sd)	36.72 (±0.50)	36.72 (±0.50)	36.78 (±0.58)	0.403
HR, mean (±sd)	81.42 (±14.71)	81.29 (±14.61)	85.24 (±16.99)	0.073
SP, mean (±sd)	136.39 (±21.54)	136.13 (±21.30)	143.89 (±26.80)	0.026
DP, mean (±sd)	78.99 (±12.52)	79.05 (±12.48)	77.32 (±13.49)	0.319
PLT, mean (±sd)	206.53 (±85.10)	206.80 (±81.52)	198.70 (±158.04)	0.687
Hb, mean (±sd)	125.19 (±21.29)	125.63 (±21.01)	112.50 (±25.18)	<0.001
RBC, mean (±sd)	4.12 (±0.73)	4.14 (±0.72)	3.65 (±0.78)	<0.001
Monocyte, mean (±sd)	0.62 (±0.30)	0.62 (±0.30)	0.58 (±0.26)	0.252
Lymphocyte, mean (±sd)	1.36 (±0.66)	1.36 (±0.66)	1.15 (±0.63)	0.011
WBC, mean (±sd)	10.94 (±4.64)	10.96 (±4.67)	10.31 (±3.83)	0.194
HDL, mean (±sd)	1.19 (±0.38)	1.19 (±0.38)	1.07 (±0.33)	0.005
LDL, mean (±sd)	2.57 (±0.85)	2.58 (±0.84)	2.27 (±0.95)	0.012
P, mean (±sd)	0.96 (±0.27)	0.96 (±0.27)	0.95 (±0.30)	0.839
K, mean (±sd)	3.90 (±0.45)	3.89 (±0.44)	4.03 (±0.58)	0.067
Na, mean (±sd)	139.28 (±4.80)	139.27 (±4.74)	139.44 (±6.19)	0.826
Ca, mean (±sd)	2.18 (±0.15)	2.18 (±0.15)	2.14 (±0.14)	0.016
ALT, mean (±sd)	27.94 (±35.85)	28.07 (±36.30)	24.19 (±18.00)	0.112
AST, mean (±sd)	32.06 (±35.74)	32.11 (±36.16)	30.58 (±19.66)	0.562
CK, mean (±sd)	385.36 (±827.65)	386.30 (±830.95)	358.04 (±730.16)	0.765
Blood glucose, mean (±sd)	6.93 (±2.79)	6.87 (±2.71)	8.63 (±4.16)	0.001
Globulin, mean (±sd)	25.72 (±4.49)	25.69 (±4.48)	26.82 (±4.66)	0.062
Total cholesterol, mean (±sd)	4.16 (±0.98)	4.17 (±0.97)	3.78 (±1.11)	0.009
Triglyceride, mean (±sd)	1.49 (±1.66)	1.49 (±1.67)	1.44 (±1.36)	0.792
GCS, mean (±sd)	13.12 (±3.40)	13.16 (±3.36)	11.71 (±4.39)	0.012
CAR, mean (±sd)	1.82 (±0.69)	1.80 (±0.67)	2.42 (±1.09)	<0.001
Gender, *n* (p%)				0.184
Female	565.00 (29.75%)	551.00 (30.01%)	14.00 (22.22%)	
Male	1,334.00 (70.25%)	1,285.00 (69.99%)	49.00 (77.78%)	
Smoking, *n* (p%)				0.257
No	1,696.00 (89.31%)	1,637.00 (89.16%)	59.00 (93.65%)	
Yes	203.00 (10.69%)	199.00 (10.84%)	4.00 (6.35%)	
Drinking, *n* (p%)				0.127
No	1,739.00 (91.57%)	1,678.00 (91.39%)	61.00 (96.83%)	
Yes	160.00 (8.43%)	158.00 (8.61%)	2.00 (3.17%)	
Hypertension, *n* (p%)				<0.001
No	1,404.00 (73.93%)	1,376.00 (74.95%)	28.00 (44.44%)	
Yes	495.00 (26.07%)	460.00 (25.05%)	35.00 (55.56%)	
Diabetes, *n* (p%)				0.002
No	1,619.00 (85.26%)	1,574.00 (85.73%)	45.00 (71.43%)	
Yes	280.00 (14.74%)	262.00 (14.27%)	18.00 (28.57%)	
Cerebral_hernia, *n* (p%)				0.166
No	1,793.00 (94.42%)	1,736.00 (94.55%)	57.00 (90.48%)	
Yes	106.00 (5.58%)	100.00 (5.45%)	6.00 (9.52%)	
Surgery, *n* (p%)				0.526
No	1,564.00 (82.36%)	1,514.00 (82.46%)	50.00 (79.37%)	
Yes	335.00 (17.64%)	322.00 (17.54%)	13.00 (20.63%)	

R, respiratory rate; HR, heart rate; T, temperature of body; SP, systolic pressure; DP, diastolic pressure; GCS, Glasgow Coma Scale; PLT, platelet; Hb, hemoglobin; RBC, red blood cell; WBC, white blood cell; HDL, high density lipoprotein; LDL, low density lipoprotein; P, phosphorus; K, kalium; Na, natrium; Ca, calcium; ALT, alanine transaminase; ASL, aspartic acid transaminase; CK, creatine kinase; CAR, creatine-albumin ratio; AHF, acute heart failure; PSM, propensity score matching; sd, standard deviation.

Continuous variables were expressed as mean ± standard deviation and categorical variables were reported as *n* (%). The *p*-value of continuous variables was calculated by independent samples *t*-test or Mann–Whitney *U* test, as appropriate; the *p*-value of categorical variables was calculated by Chi-square test or Fisher’s exact test, as appropriate.

Biochemical and metabolic differences further differentiated TBI patients with AHF. They had lower high-density lipoprotein (HDL) (1.07 ± 0.33 mmol/L vs. 1.19 ± 0.38 mmol/L, *p* = 0.005) and low-density lipoprotein (LDL) levels (2.27 ± 0.95 mmol/L vs. 2.58 ± 0.84 mmol/L, *p* = 0.012), along with a higher CAR (2.42 ± 1.09 vs. 1.80 ± 0.67, *p* < 0.001). Patients with AHF also had lower GCS scores (11.71 ± 4.39 vs. 13.16 ± 3.36, *p* = 0.012), reflecting more severe neurological injury. Moreover, hypertension (55.56% vs. 25.05%, *p* < 0.001) and diabetes (28.57% vs. 14.27%, *p* = 0.002) were significantly more prevalent in the AHF group.

### 3.2 Covariate balance after PSM

**Figure 2:**
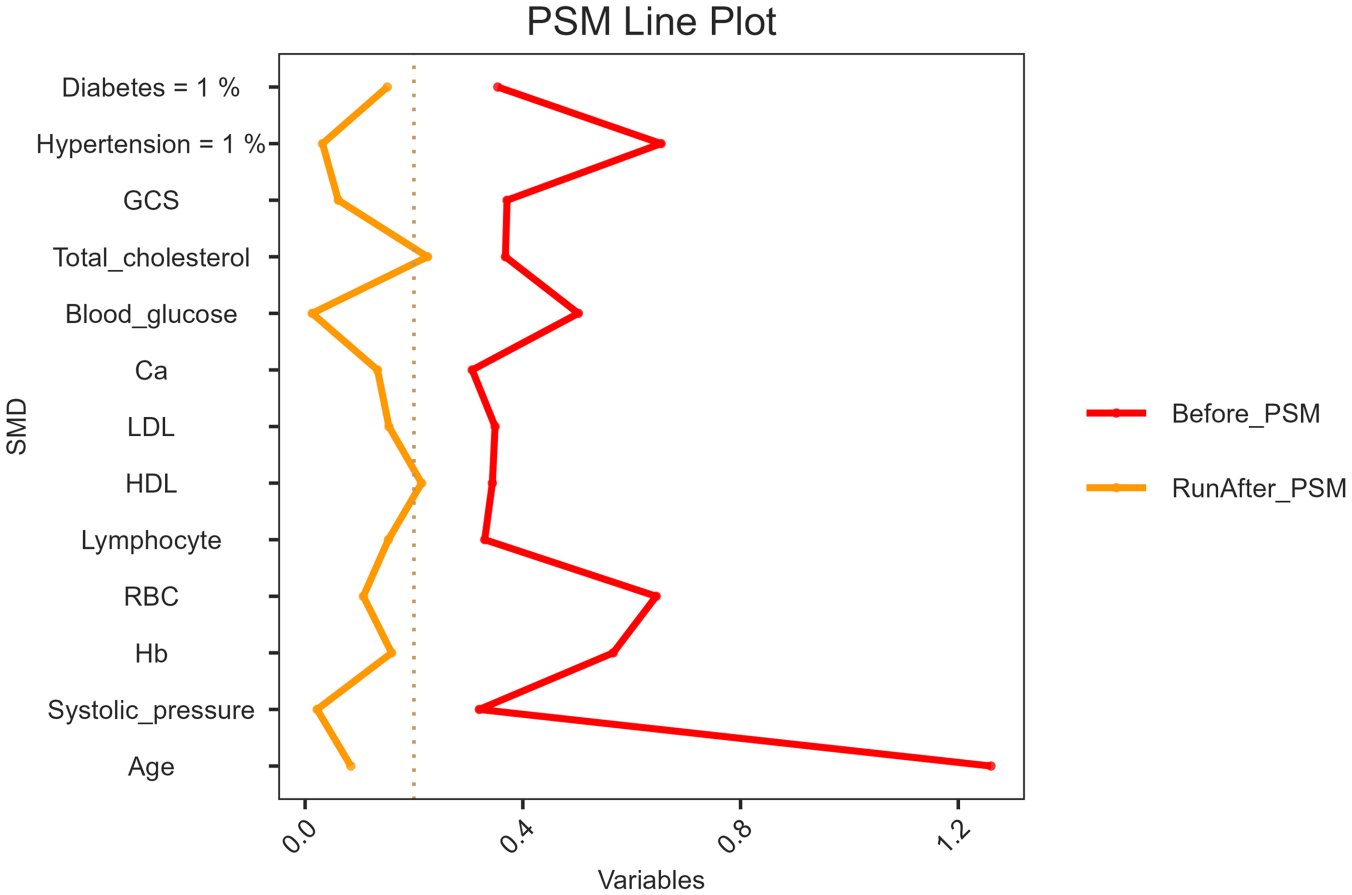
Propensity score matching (PSM) line plot of standardized mean differences before and after matching. This line plot illustrates the standardized mean differences (SMD) of key variables before (red line) and after (yellow line) propensity score matching (PSM). The variables include demographic, clinical, and biochemical characteristics. The vertical dashed line at SMD = 0.2 represents the threshold for acceptable balance. After PSM, the SMDs of most variables were reduced below 0.2, indicating improved covariate balance between the matched groups.

Figure 2 illustrates the improvement in covariate balance after PSM. Before matching, standardized mean differences (SMDs) for several variables, including age, systolic blood pressure, hemoglobin, and red blood cell count, were above the acceptable threshold of 0.2, indicating significant imbalance between the groups. This is represented by the red line, which highlights substantial differences in key variables before matching. After PSM, the yellow line shows a notable reduction in SMDs, with most variables falling below the 0.2 threshold, indicating a substantial improvement in balance. Key variables that were highly imbalanced, such as age and systolic blood pressure, were effectively adjusted, ensuring comparability between the groups. This indicates that PSM effectively minimized confounding factors, thereby enhancing the validity of subsequent analyses.

### 3.3 Baseline demographic and clinical characteristics after PSM

After PSM, the baseline demographic and clinical characteristics between the AHF and non-AHF groups following TBI were well-balanced, with most variables showing no significant differences (Table 2). The mean age of participants was similar between the groups (74.51 ± 11.68 years, *p* = 0.641), as were GCS scores (11.73 ± 4.24, *p* = 0.736) and systolic blood pressure (143.60 ± 25.35 mmHg, *p* = 0.905). Hemoglobin levels, red blood cell count, and other hematological parameters also showed no significant differences. However, the CAR was significantly higher in the AHF group compared to the non-AHF group (2.39 ± 1.05 vs. 2.05 ± 0.70, *p* = 0.038), suggesting its potential association with AHF. Other biochemical variables, including lipid profiles, blood glucose, and electrolytes, showed no statistical differences between the groups. These results suggest that the PSM process achieved a balanced comparison across most variables while retaining differences in CAR, which may have clinical relevance.

**Table 2:** Baseline demographic and clinical characteristics of the study population after PSM.

Variable	Overall	Non-AHF	AHF	*p*-value
	*N* = 124	*N* = 62	*N* = 62	
Age, mean (±sd)	74.51 (±11.68)	75.00 (±10.80)	74.02 (±12.56)	0.641
GCS, mean (±sd)	11.73 (±4.24)	11.60 (±4.23)	11.85 (±4.28)	0.736
Time_onset, mean (±sd)	236.60 (±799.26)	197.45 (±681.97)	275.74 (±905.48)	0.588
R, mean (±sd)	19.65 (±4.54)	19.11 (±4.91)	20.19 (±4.11)	0.187
T, mean (±sd)	36.77 (±0.54)	36.75 (±0.49)	36.78 (±0.58)	0.790
HR, mean (±sd)	83.52 (±15.67)	81.23 (±14.56)	85.81 (±16.51)	0.104
SP, mean (±sd)	143.60 (±25.35)	143.32 (±23.78)	143.87 (±27.02)	0.905
DP, mean (±sd)	77.95 (±14.23)	78.44 (±14.98)	77.47 (±13.54)	0.707
PLT, mean (±sd)	200.85 (±144.20)	202.29 (±128.73)	199.42 (±159.23)	0.912
Hb, mean (±sd)	114.74 (±22.46)	116.53 (±19.49)	112.95 (±25.12)	0.378
RBC, mean (±sd)	3.70 (±0.71)	3.74 (±0.64)	3.67 (±0.78)	0.552
Monocyte, mean (±sd)	0.58 (±0.27)	0.58 (±0.27)	0.58 (±0.26)	0.995
Lymphocyte, mean (±sd)	1.11 (±0.61)	1.07 (±0.59)	1.16 (±0.63)	0.396
WBC, mean (±sd)	10.38 (±4.20)	10.52 (±4.60)	10.23 (±3.80)	0.702
HDL, mean (±sd)	1.11 (±0.37)	1.15 (±0.41)	1.07 (±0.33)	0.237
LDL, mean (±sd)	2.33 (±0.91)	2.40 (±0.87)	2.26 (±0.95)	0.392
P, mean (±sd)	0.93 (±0.29)	0.90 (±0.30)	0.96 (±0.28)	0.203
K, mean (±sd)	3.95 (±0.56)	3.87 (±0.54)	4.02 (±0.58)	0.137
Na, mean (±sd)	139.50 (±5.42)	139.51 (±4.51)	139.49 (±6.23)	0.978
Ca, mean (±sd)	2.14 (±0.14)	2.15 (±0.15)	2.14 (±0.14)	0.462
ALT, mean (±sd)	25.08 (±24.36)	25.90 (±29.44)	24.27 (±18.14)	0.712
AST, mean (±sd)	30.77 (±19.03)	31.52 (±18.90)	30.01 (±19.28)	0.661
CK, mean (±sd)	332.49 (±596.63)	306.15 (±417.77)	358.82 (±736.10)	0.625
Blood glucose, mean (±sd)	8.44 (±3.75)	8.41 (±3.56)	8.46 (±3.95)	0.943
Globulin, mean (±sd)	26.41 (±4.94)	25.88 (±5.24)	26.93 (±4.61)	0.240
Total cholesterol, mean (±sd)	3.89 (±1.03)	4.01 (±0.93)	3.78 (±1.12)	0.213
Triglyceride, mean (±sd)	1.54 (±1.47)	1.63 (±1.58)	1.46 (±1.37)	0.522
CAR, mean (±sd)	2.22 (±0.91)	2.05 (±0.70)	2.39 (±1.05)	0.038
Gender, *n* (p%)				0.832
Female	29.00 (23.39%)	15.00 (24.19%)	14.00 (22.58%)	
Male	95.00 (76.61%)	47.00 (75.81%)	48.00 (77.42%)	
Smoking, *n* (p%)				0.510
No	114.00 (91.94%)	56.00 (90.32%)	58.00 (93.55%)	
Yes	10.00 (8.06%)	6.00 (9.68%)	4.00 (6.45%)	
Drinking, *n* (p%)				0.403
No	118.00 (95.16%)	58.00 (93.55%)	60.00 (96.77%)	
Yes	6.00 (4.84%)	4.00 (6.45%)	2.00 (3.23%)	
Hypertension, *n* (p%)				0.857
No	57.00 (45.97%)	29.00 (46.77%)	28.00 (45.16%)	
Yes	67.00 (54.03%)	33.00 (53.23%)	34.00 (54.84%)	
Diabetes, *n* (p%)				0.402
No	94.00 (75.81%)	49.00 (79.03%)	45.00 (72.58%)	
Yes	30.00 (24.19%)	13.00 (20.97%)	17.00 (27.42%)	
Cerebral_hernia, *n* (p%)				0.544
No	112.00 (90.32%)	55.00 (88.71%)	57.00 (91.94%)	
Yes	12.00 (9.68%)	7.00 (11.29%)	5.00 (8.06%)	
Surgery, *n* (p%)				0.390
No	96.00 (77.42%)	46.00 (74.19%)	50.00 (80.65%)	
Yes	28.00 (22.58%)	16.00 (25.81%)	12.00 (19.35%)	

R, respiratory rate; HR, heart rate; T, temperature of body; SP, systolic pressure; DP, diastolic pressure; GCS, Glasgow Coma Scale; PLT, platelet; Hb, hemoglobin; RBC, red blood cell; WBC, white blood cell; HDL, high density lipoprotein; LDL, low density lipoprotein; P, phosphorus; K, kalium; Na, natrium; Ca, calcium; ALT, alanine transaminase; ASL, aspartic acid transaminase; CK, creatine kinase; CAR, creatine-albumin ratio; AHF, acute heart failure; PSM, propensity score matching; sd, standard deviation.

Data are presented as mean ± standard deviation or *n* (%). Between-group comparisons after matching were conducted with paired *t*-tests or Wilcoxon signed-rank tests for continuous variables and McNemar’s test or conditional logistic regression for categorical variables, as appropriate.

### 3.4 Predictive value of CAR for AHF after TBI in pre- and post-PSM

Table 3 compares the performance of CAR in predicting AHF after TBI before and after PSM using logistic regression and ROC analysis. In the pre-PSM logistic regression, CAR showed strong predictive ability in single-factor analysis (*p* < 0.001, OR: 1.885, 95% CI: 1.534–2.318) and multiple-factor analysis (*p* = 0.027, OR: 1.324, 95% CI: 1.032–1.698). However, after PSM, the predictive power was reduced, with a marginally significant single-factor analysis (*p* = 0.047, OR: 1.597, 95% CI: 1.007–2.532) and multiple-factor analysis (p = 0.047, OR: 1.597, 95% CI: 1.007–2.532).

**Table 3:** Comparison of the predictive performance of CAR for AHF before and after PSM.

CAR	Before PSM	After PSM
Logistic regression analysis		
Single factor analysis		
*P*-value	*P* < 0.001	*P* = 0.047
OR (95%CI)	1.885 (1.534–2.318)	1.597 (1.007–2.532)
Multiple-factor analysis		
*P*-value	*P* = 0.027	*P* = 0.047
OR (95%CI)	1.324 (1.032–1.698)	1.597 (1.007–2.532)
ROC curve		
*P*-value	*P* < 0.001	*P* = 0.050
AUC	0.721	0.599
Yuden index J	0.418	0.194
Sensitivity	71.43%	70.97%
Specificity	70.37%	48.39%

CAR, creatine-albumin ratio; AHF, acute heart failure; PSM, propensity score matching; OR, odds ratio; 95%CI, 95% confidence interval; ROC, receiver operating characteristic; AUC, area under the curve.

Logistic regression analyses (univariable and multivariable) and receiver operating characteristic (ROC) curve analysis were used to determine the predictive value of creatinine-to-albumin ratio (CAR). Odd ratios (OR) and area under the curve (AUC) are reported with corresponding *p*-values.

**Figure 3:**
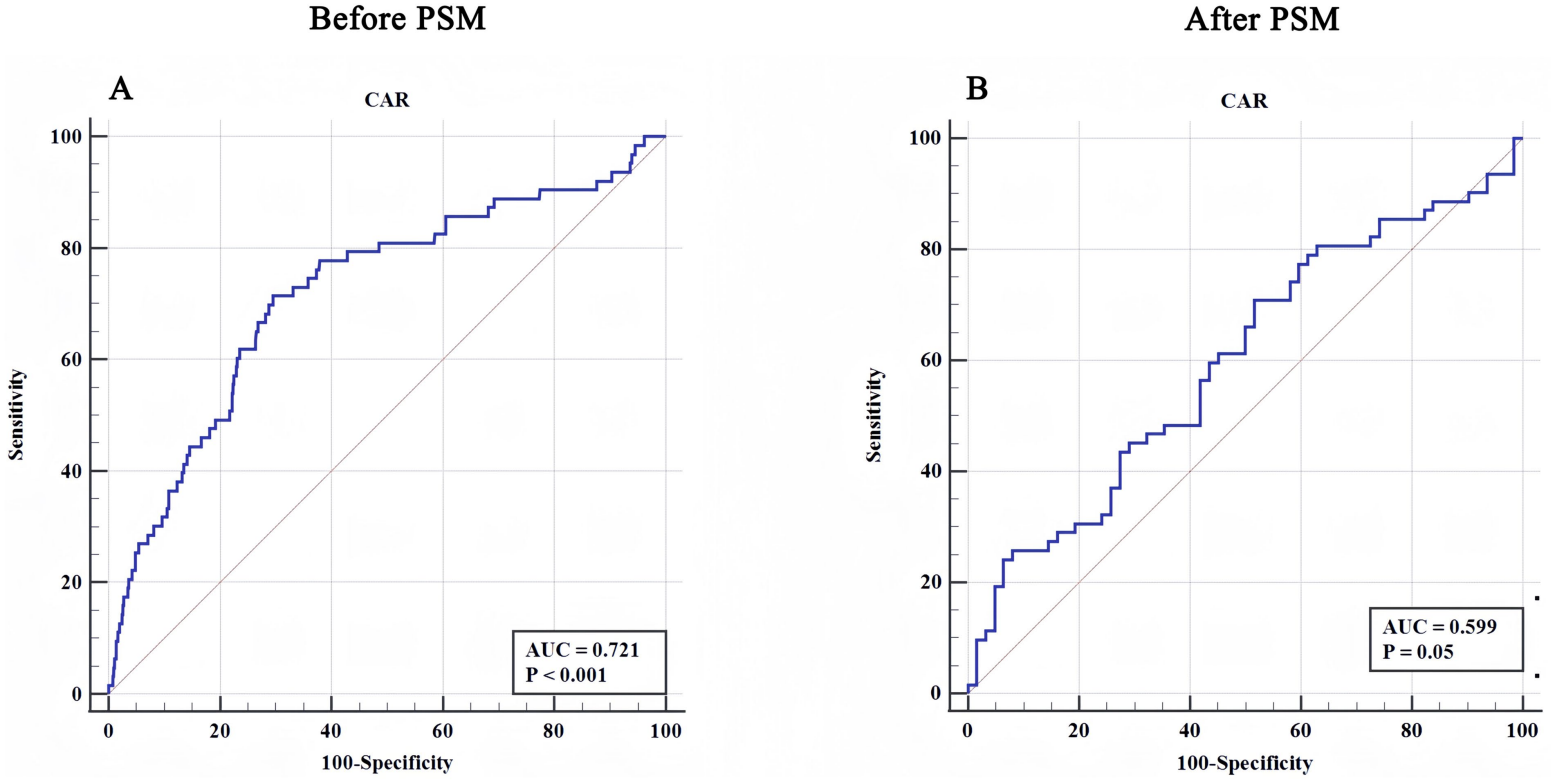
ROC curves for CAR in predicting AHF in pre- and post-PSM. Panel **(A)** shows the receiver operating characteristic (ROC) curve for the creatinine-to-albumin ratio (CAR) before PSM. The area under the curve (AUC) is 0.721, with a statistically significant *p*-value (<0.001), indicating strong discriminatory ability. Panel **(B)** displays the ROC curve for CAR after PSM, with an AUC of 0.599 and a *p*-value of 0.05, suggesting moderate predictive performance. These results highlight the potential of CAR as a predictive biomarker for AHF.

The ROC analysis revealed a decrease in predictive performance Pre- and Post-PSM. Figure 3 illustrates the diagnostic performance of the CAR in predicting AHF through ROC analysis. In the cohort (Figure 3A), before PSM, CAR demonstrated strong predictive ability, with an AUC of 0.721 and a statistically significant *p* < 0.001, with a Youden index of 0.418, sensitivity of 71.43%, and specificity of 70.37%. This result underscores CAR as a reliable biomarker for distinguishing patients with AHF after TBI from those without. After PSM (Figure 3B), CAR showed moderate predictive performance, with an AUC of 0.599 and a *p*-value of 0.050, with a lower Youden index of 0.194 and a reduced specificity of 48.39%. Although the predictive ability after PSM was less pronounced, the results still suggest some potential for CAR as a marker in specific clinical contexts. Overall, these findings highlight the utility of CAR as a diagnostic tool for AHF, particularly in larger cohorts (Table 3).

### 3.5 CAR multiple model regression analysis

Table 4 presents the results of multiple regression models evaluating the association between the CAR and AHF after TBI. In the unadjusted Model-1, CAR showed a strong and significant association with AHF, with an OR of 1.885 (95% CI: 1.534–2.318, *p* < 0.001). After adjusting for confounding factors, including gender, age, smoking, alcohol consumption, hypertension, and diabetes in Model-2, the association remained significant, though slightly attenuated, with an OR of 1.480 (95% CI: 1.157–1.891, *p* = 0.002). These results indicate that CAR is an independent predictor of AHF in patients with TBI, even after adjusting for key demographic and clinical variables.

**Table 4:** Multiple LR analysis of the association between CAR and AHF after TBI.

Exposure	Model-1		Model-2	
	OR (95%CI)	*P*-value	OR (95%CI)	*P*-value
CAR	1.885 (1.534,2.318)	<0.001	1.480 (1.157,1.891)	0.002

Model-1: non-adjusted.

Model-2: adjust for: gender, age, smoking, drinking, hypertension, diabetes.

CAR, creatine-albumin ratio; LR, logistic regression; TBI, traumatic brain injury.

*P*-values and odd ratios (OR) with 95% confidence intervals were calculated by logistic regression.

### 3.6 Non-linear association between CAR and AHF after TBI

**Figure 4:**
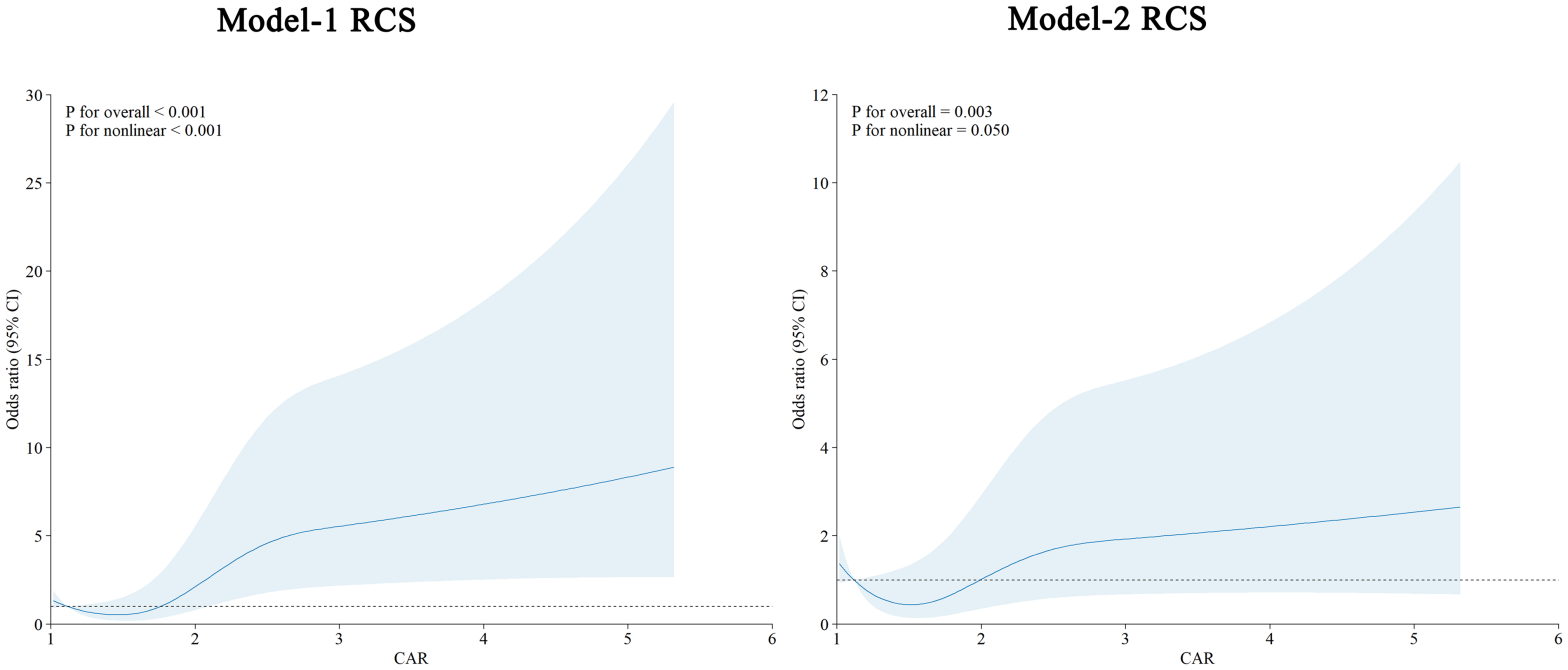
Restricted cubic spline (RCS) curves for the association between CAR and AHF. Model-1 shows the unadjusted RCS curve, revealing a stronger overall association (*p* < 0.001) and significant non-linearity (*p* < 0.001). Model-2 displays the adjusted RCS curve, which controls for gender, age, smoking, drinking, hypertension, and diabetes, with a significant overall association (*p* = 0.003) and marginal nonlinearity (*p* = 0.050). The curves illustrate that higher creatinine-to-albumin ratio (CAR) values are consistently associated with increased odds of AHF. Shaded areas represent the 95% confidence intervals.

Figure 4 illustrates the RCS analysis of the association between the CAR and the odds of AHF after TBI in unadjusted (Model-1) and adjusted (Model-2) logistic regression models. In the unadjusted Model-1, the association was stronger and more nonlinear (*p* < 0.001 for both overall association and non-linearity). In the adjusted Model-2, which accounts for confounding factors such as gender, age, smoking, alcohol consumption, hypertension, and diabetes, CAR remained significantly associated with AHF (*p* = 0.003), with marginal evidence of nonlinearity (*p* = 0.050). The adjusted curve indicates a steeper increase in OR with rising CAR values, highlighting that the risk of AHF escalates disproportionately at higher CAR levels. These findings suggest that CAR is a robust and independent predictor of AHF after TBI, with a nonlinear relationship indicating an amplified risk at higher CAR values.

### 3.7 CAR group trend test

Table 5 presents the trend analysis of CAR groups in relation to the odds of AHF after TBI. In the unadjusted Model-1, a significant increasing trend across CAR quartiles was observed, with the highest quartile (Q4, CAR: 2.022–7.968) demonstrating substantially elevated odds of AHF (OR: 5.980, 95% CI: 2.647–13.511, *p* < 0.001). The group trend test further confirmed this significant association, with an OR of 7.102 (95% CI: 3.725–13.540, *p* < 0.001), indicating a strong positive trend in the risk of AHF as CAR levels increased. After adjusting for confounders such as gender, age, smoking, alcohol consumption, hypertension, and diabetes in Model-2, the trend remained significant (OR for trend: 3.268, 95% CI: 1.616–6.610, *p* < 0.001). However, the strength of the association was attenuated. While the adjusted odds for Q4 were elevated (OR: 2.357, 95% CI: 0.959–5.793), they did not reach statistical significance (*p* = 0.062). These results highlight a robust positive trend between higher CAR levels and increased odds of AHF, reinforcing CAR as a valuable predictor, even after adjusting for potential confounders.

**Table 5:** Trend analysis of AHF risk across quartiles of the CAR.

Exposure	Model-1		Model-2	
CAR group	OR (95%CI)	*P*-value	OR (95%CI)	*P*-value
Q1 (1.002–1.403)		1.000		1.000
Q2 (1.404–1.662)	0.719 (0.227,2.281)	0.575	0.540 (0.166,1.761)	0.307
Q3 (1.667–2.021)	1.718 (0.670,4.402)	0.260	1.036 (0.384,2.797)	0.492
Q4 (2.022–7.968)	5.980 (2.647,13.511)	<0.001	2.357 (0.959,5.793)	0.062
GPR group trend	7.102 (3.725,13.540)	<0.001	3.268 (1.616,6.610)	<0.001

Model-1: non-adjusted.

Model-2: adjust for: gender, age, smoking, drinking, hypertension, diabetes.

CAR, creatine-albumin ratio; AHF, acute heart failure.

The *p* for trend was calculated by treating the CAR quartiles as an ordinal variable in the logistic regression model. Model 1 was unadjusted; Model 2 was adjusted for gender, age, smoking, drinking, hypertension and diabetes.

## 4 Discussion

In our study, CAR can be an independent predictor of AHF occurrence among the patients with TBI. CAR can differentiate AHF occurrence with high discriminative performance (AUC = 0.721), in addition to its independent correlation with AHF occurrence by univariate and multivariate logistic regression analyses before PSM. The independent association between CAR and AHF occurrence in TBI patients can be attenuated after the rigorous matching of the baseline covariates by PSM, and the relatively lower predictive performance of CAR (AUC = 0.599) can be due to its attenuation after matching for significant confounding factors. This study used a RCS to graphically examine the association of CAR with AHF occurrence. The nonlinear dose–response relationship with an OR for AHF in the CAR third quartile of 2.68 compared to the lowest quartile (OR for Q4 vs. Q1 = 5.34) was observed. In addition, the use of a trend test for the increasing quartiles of CAR indicated a significant trend in the risk of AHF occurrence (*P* for trend < 0.001), suggesting a graded relationship between CAR and the risk of AHF occurrence. Our findings are novel in the prediction of AHF with the use of CAR in TBI patients.

Our study contributes to the existing literature on biomarkers for post-TBI complications in several key ways. Previous research has extensively explored the role of established cardiac biomarkers, such as BNP and troponin, in TBI populations. However, as noted by (1) BNP’s utility in TBI is complicated by its release from injured brain tissue, independent of cardiac function, potentially limiting its specificity for AHF in this population (1). Troponin elevation in TBI has also been reported to often indicate neurogenic stress-induced cardiac injury rather than acute coronary syndromes, as observed in a cohort study by Cai et al. (7). CAR, in contrast, is a composite biomarker reflecting renal function, systemic inflammation, and nutritional status, all of which are pathophysiological processes implicated in the systemic response to severe TBI and multi-organ dysfunction. While prior biomarker investigations in TBI have primarily centered on neurological injury and prognosis, our study specifically focuses on the association between CAR and AHF, a frequent yet under-investigated systemic complication. Moreover, our study overcomes the limitation of single-timepoint analyses by employing rigorous methods, including PSM to reduce confounding bias and RCS to accurately model non-linear associations, thereby strengthening the evidence for the risk stratification role of CAR in AHF among TBI patients.

The present findings support the role of CAR as a valid, synergistic, multimarker by incorporating the multiple pathophysiologic processes that mediate the TBI-AHF relationship. The pathophysiologic processes by which this association is likely to be mediated include a cluster of systemic inflammation, increased oxidative stress, and metabolic derangement. An upregulation of pro-inflammatory mediators such as IL-6 and TNF-*α* following TBI causes a systemic inflammatory response. In turn, this response, coupled with oxidative stress, can suppress hepatic albumin production and lead to renal dysfunction, as indicated by increased serum creatinine levels (18–20). In addition, this inflammatory cascade and oxidative stress, can directly cause myocardial injury and dysfunction, increased vascular permeability, and afterload, all of which can accelerate albumin breakdown (21–23). Moreover, hypoalbuminemia and increased creatinine levels may also reduce antioxidant reserves, and hence increase cardiomyocyte susceptibility to oxidative injury, and possibly mitochondrial dysfunction leading to decreased cardiac contractility, respectively (24–26). In sum, the CAR incorporates the impact of these two simultaneous processes: the pathophysiological stress and catabolic state associated with renal dysfunction, and the systemic inflammatory response, which can lead to cardiac damage (27, 28). Notably, we observed a non-linear relationship between CAR and risk of AHF such that beyond a certain point, the relative risk of AHF increased exponentially. This may point to the presence of a pathophysiologic threshold, beyond which the aggregate effect of these processes leading to cardiac damage increases, in a manner not explained by any of the single biomarkers such as the traditional laboratory markers. Taken together, these mechanistic insights and their alignment with the observed risk patterns support the biological plausibility of CAR as a marker of risk and help to distinguish its proposed predictive utility from the traditional, unidirectional markers.

## 5 Limitations and future perspectives

This study provides valuable insights into the association between the CAR and AHF after TBI; however, it has several limitations that must be considered. First, as a single-center retrospective study, it is subject to selection bias, limiting the generalizability of the findings to other populations and healthcare settings (29). Second, although PSM and multivariate analyses were used to adjust for confounders, residual confounding cannot be entirely excluded. Third, the study focused solely on CAR as a biomarker and did not evaluate other potential predictors or mediators, such as cardiac-specific biomarkers like troponins or BNP (30). Lastly, the study did not include longitudinal measurements of CAR, which would have provided dynamic insights into its role in the progression of AHF over time.

To address these limitations, future studies should: (1) Conduct multicenter, prospective cohort studies to enhance generalizability and minimize selection bias. (2) Furthermore, the exclusion of patients who developed in-hospital AKI poses a potential for selection bias, as this group remains susceptible to AHF, thereby restricting the applicability of our results to all TBI patients. (3) The limited number of AHF events in our cohort may affect the precision of our estimates and warrants further validation in larger studies. (4) Include additional biomarkers, such as troponins and BNP, to evaluate their combined predictive value with CAR for AHF. (5) Implement longitudinal analyses of CAR to explore its dynamic changes over time and their correlation with clinical outcomes. (6) Investigate the mechanisms linking CAR to inflammation, oxidative stress, and cardiac dysfunction using experimental models. (7) Assess the impact of targeted interventions, such as fluid management, anti-inflammatory treatments, and nutritional support, on CAR and AHF outcomes in TBI patients.

## 6 Conclusion

This study highlights the potential of the CAR as a valuable biomarker for predicting AHF in patients with TBI. By reflecting renal dysfunction, systemic inflammation, and nutritional status, CAR offers a practical tool for early risk stratification in TBI patients. However, its clinical application requires further validation through multicenter, prospective studies to confirm its utility across diverse populations and healthcare settings. Future research should also explore the integration of CAR with other biomarkers to enhance predictive accuracy and clinical utility. Investigating the impact of targeted interventions, including nutritional support and anti-inflammatory therapies, on CAR and associated outcomes may further enhance its clinical relevance and improve outcomes in TBI patients at risk for AHF.

## Data Availability

The raw data supporting the conclusions of this article will be made available by the authors, without undue reservation.
